# Supplementary material for: Changing role of coral reef marine reserves in a warming climate
Source: Nat Commun. 2020 Apr 24;11:2000. doi: 10.1038/s41467-020-15863-z (PMC7181733; doi:10.1038/s41467-020-15863-z)
Supplement: Supplementary file 1 — Supplementary Information [file 41467_2020_15863_MOESM1_ESM.pdf]

Supplementary Information

The Changing Role of Coral Reef Marine Reserves in a Warming Climate

Graham et al.

Supplementary Figures

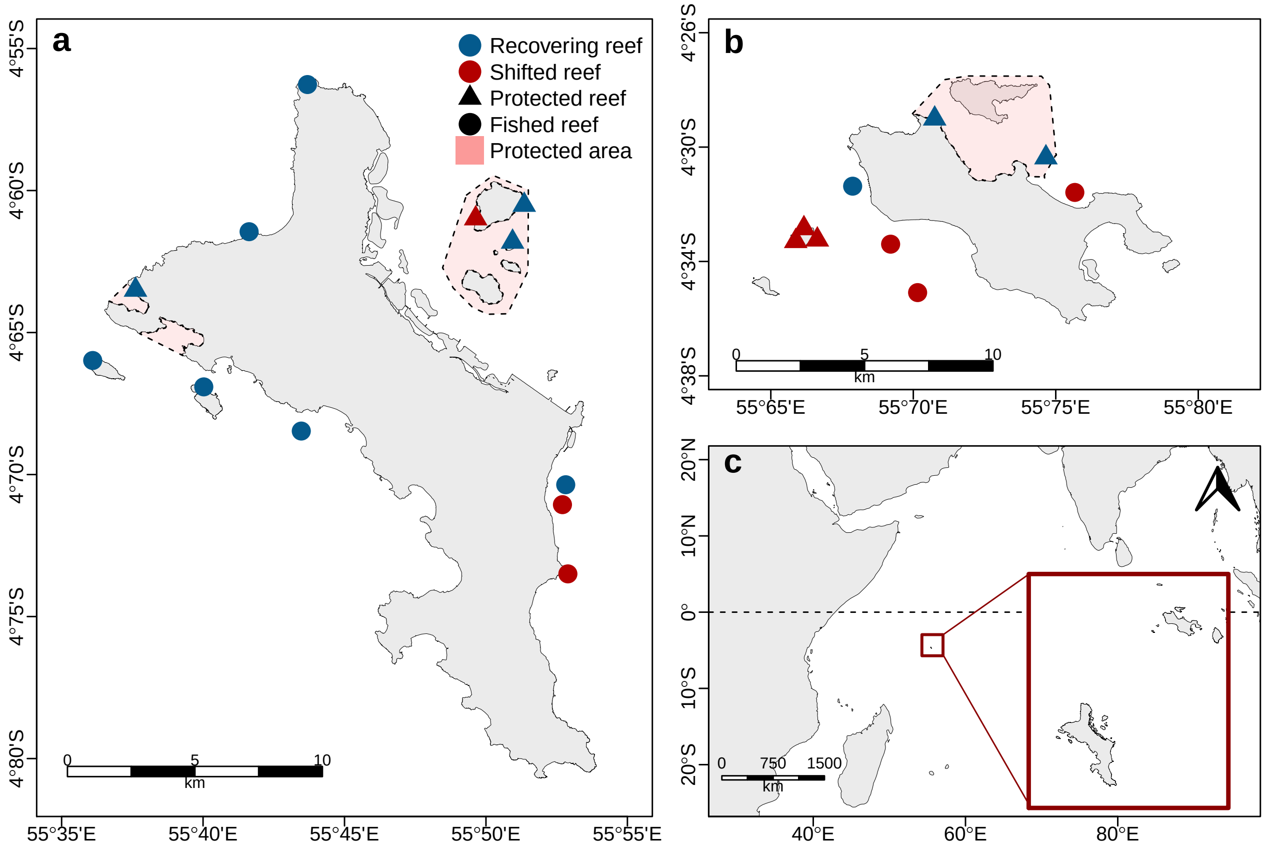

Supplementary Figure 1. Map of study sites on Mahe (a) and Praslin (b), and inner Seychelles location in the Western Indian Ocean (c). Recovering reefs are blue and regime-shifted reefs are red. Circles indicated fished reefs and triangles indicated protected reefs in marine reserves (pink shading).

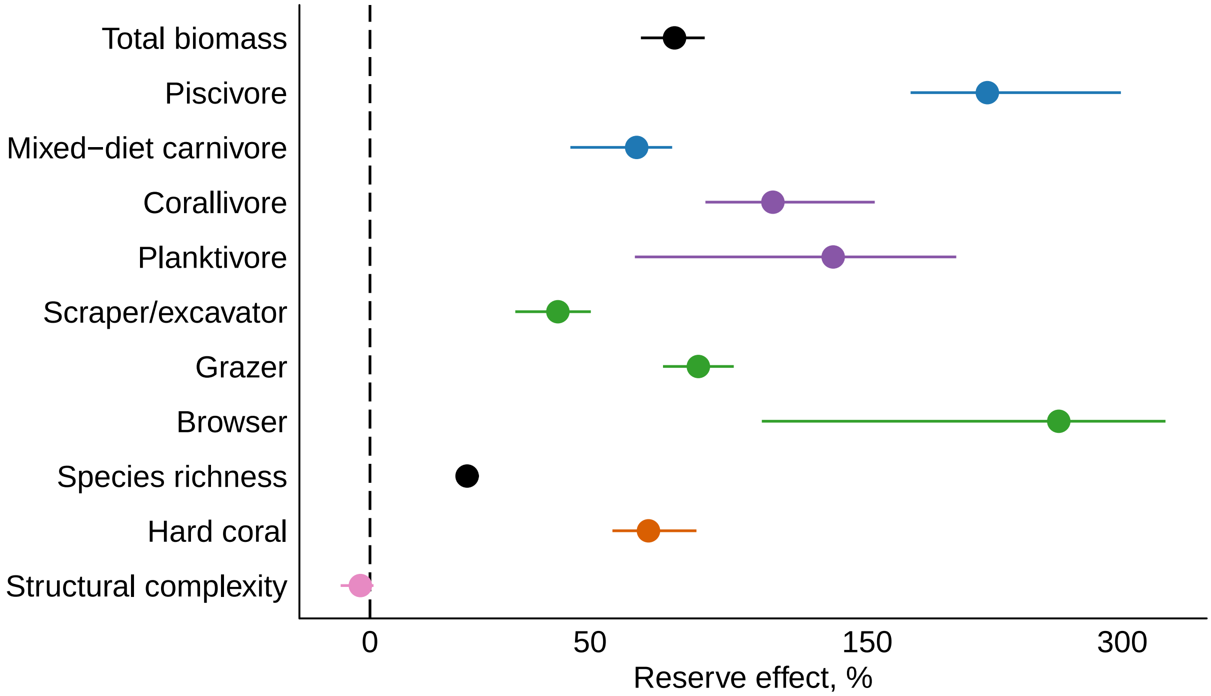

Supplementary Figure 2. Marine reserve effects before mass coral bleaching. Points are the reserve response ratio(log(reserve/fished)) for each ecological variable on nine reef reserves relative to 12 fished reefs (0% = equal values on reserve and fished reefs). Error bars are drop-one jackknife confidence intervals showing the variance in reserve effects attributable to individual reef sites. Pre-bleaching macroalgal cover was negligible at all 21 sites and is excluded here (mean = 1.2% ± 5.66 standard error, SE). Corresponding raw reserve effect values provided in Figure 1. Source data are provided as a Source Data file.

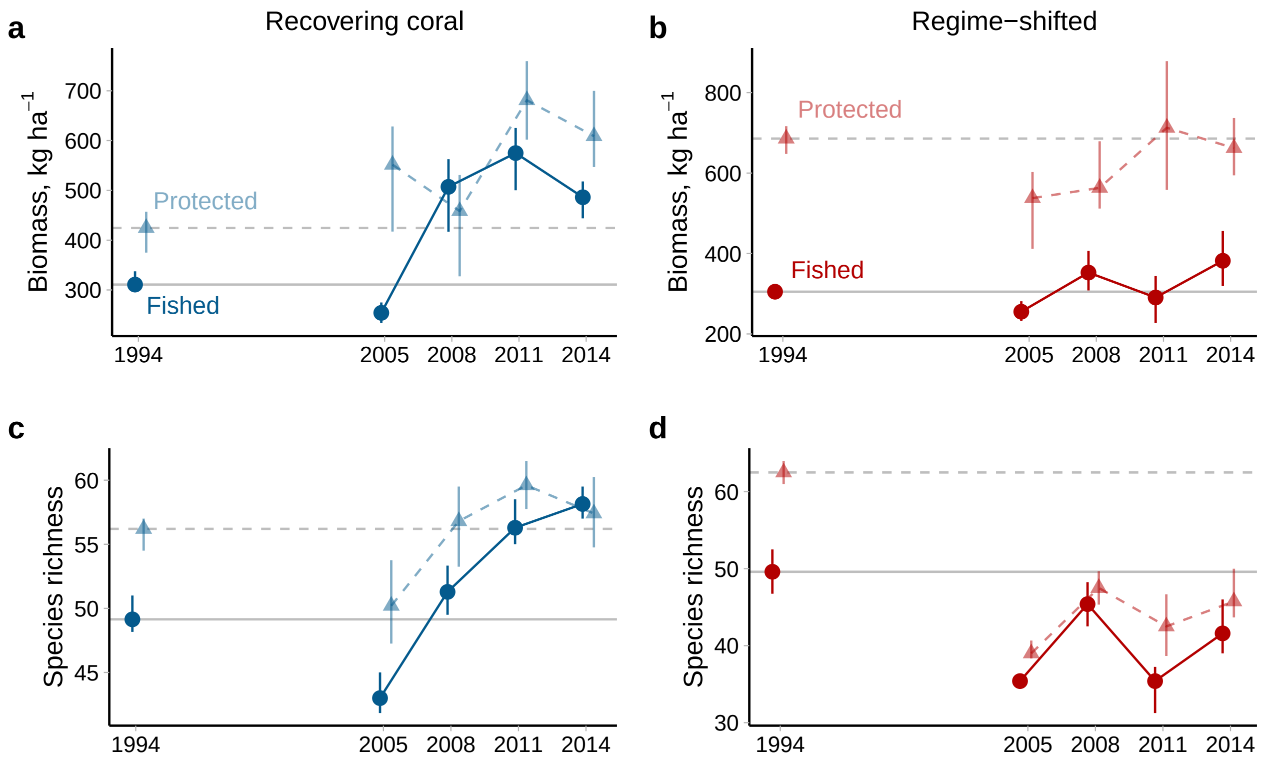

Supplementary Figure 3. Fish biomass (a,b) and species richness (c,d) from 1994-2014 at each reef in protected (dashed lines and triangles) and fished areas (solid lines and circles), for recovering (blue) and regime-shifted (red) habitats (n sites = 7 fished-recovering, 5 protected-recovering, 5 fished-shifted, 4 protected-shifted). Points are mean biomass or richness, with drop-one jacknife confidence limits. Source data are provided as a Source Data file.

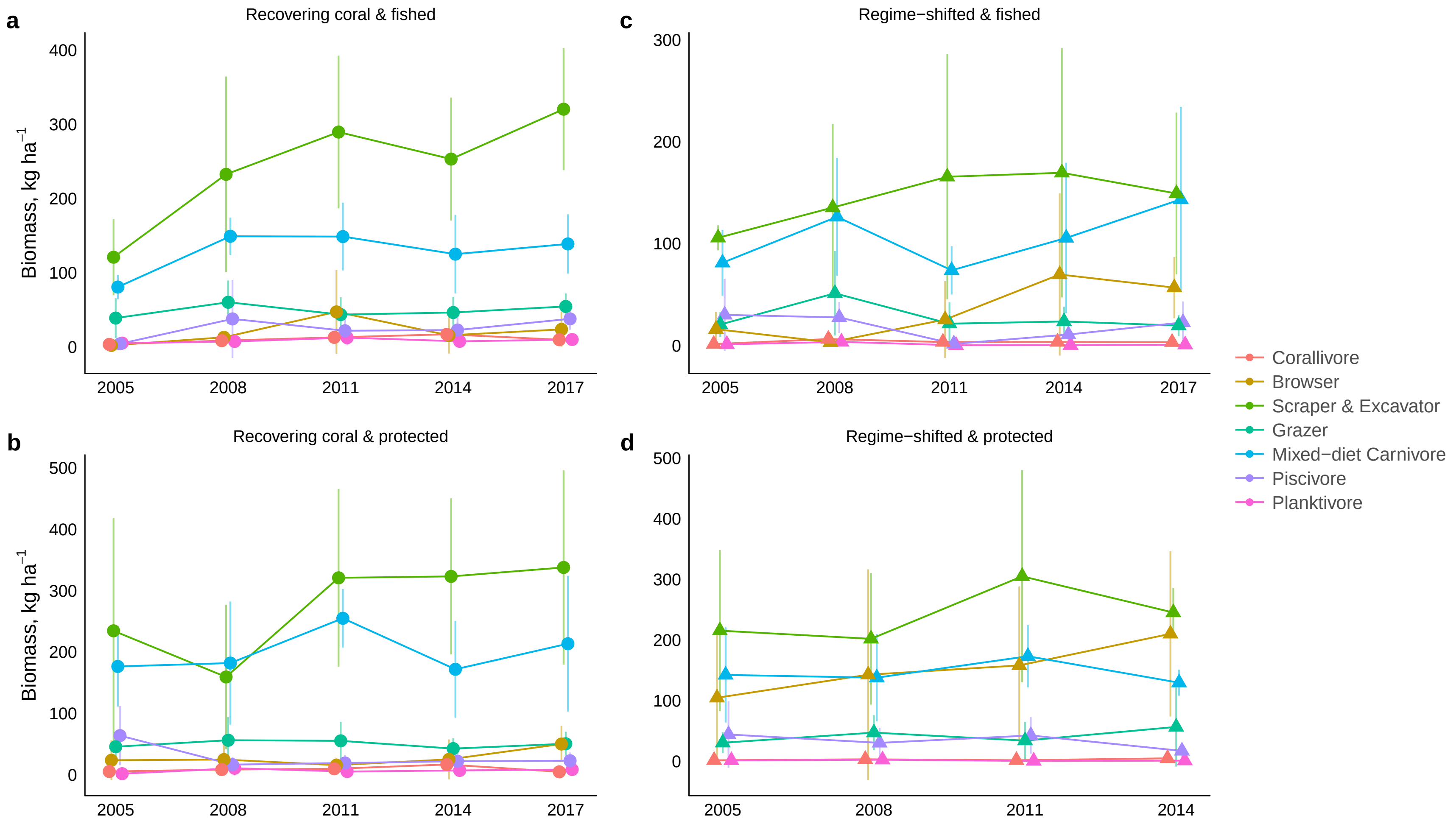

Supplementary Figure 4. Post-bleaching fish biomass of functional feeding groups from 2005-2014. Points are observed fish biomass (± 2 SE) for each functional feeding group (colours), in reefs that were recovering and fished (a, n = 7), recovering and protected (b, n = 5), regime-shifted and fished (c, n = 5) and regime-shifted and protected (d, n = 4).

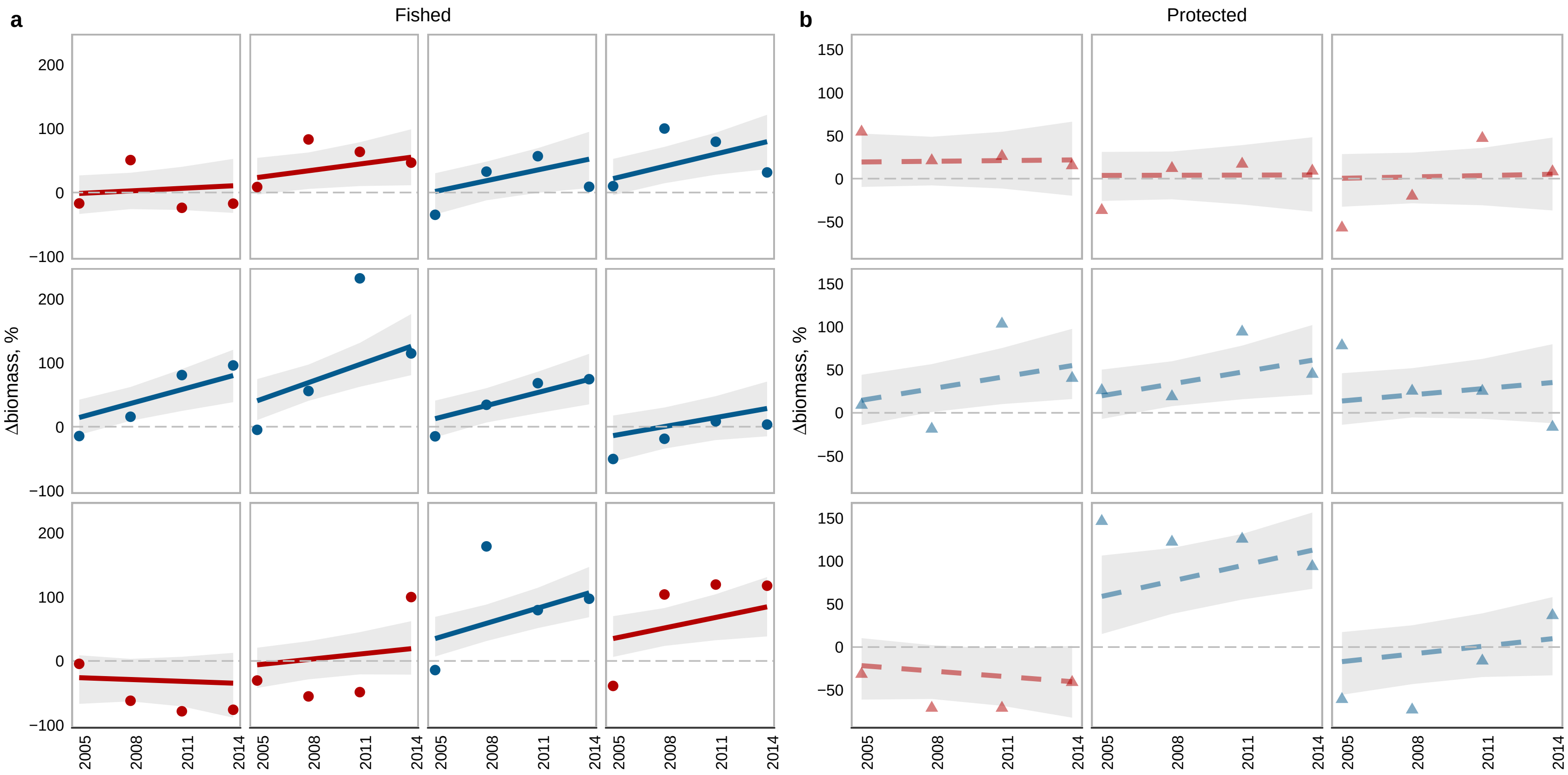

Supplementary Figure 5. Post-bleaching marine reserve effects on fish biomass of fish assemblages from 2005-2014 for individual reef sites. Panels show the recovery of biomass relative to pre-bleaching levels (y = 0, dashed grey line), for fished (a) and protected (b) reefs. Lines are posterior median predictions for each reef site (± 95% certainty intervals), for recovering (blue) and regime-shifted (red) habitats. Points are observed data (n sites = 7 fished-recovering, 5 protected-recovering, 5 fished-shifted, 4 protected-shifted).

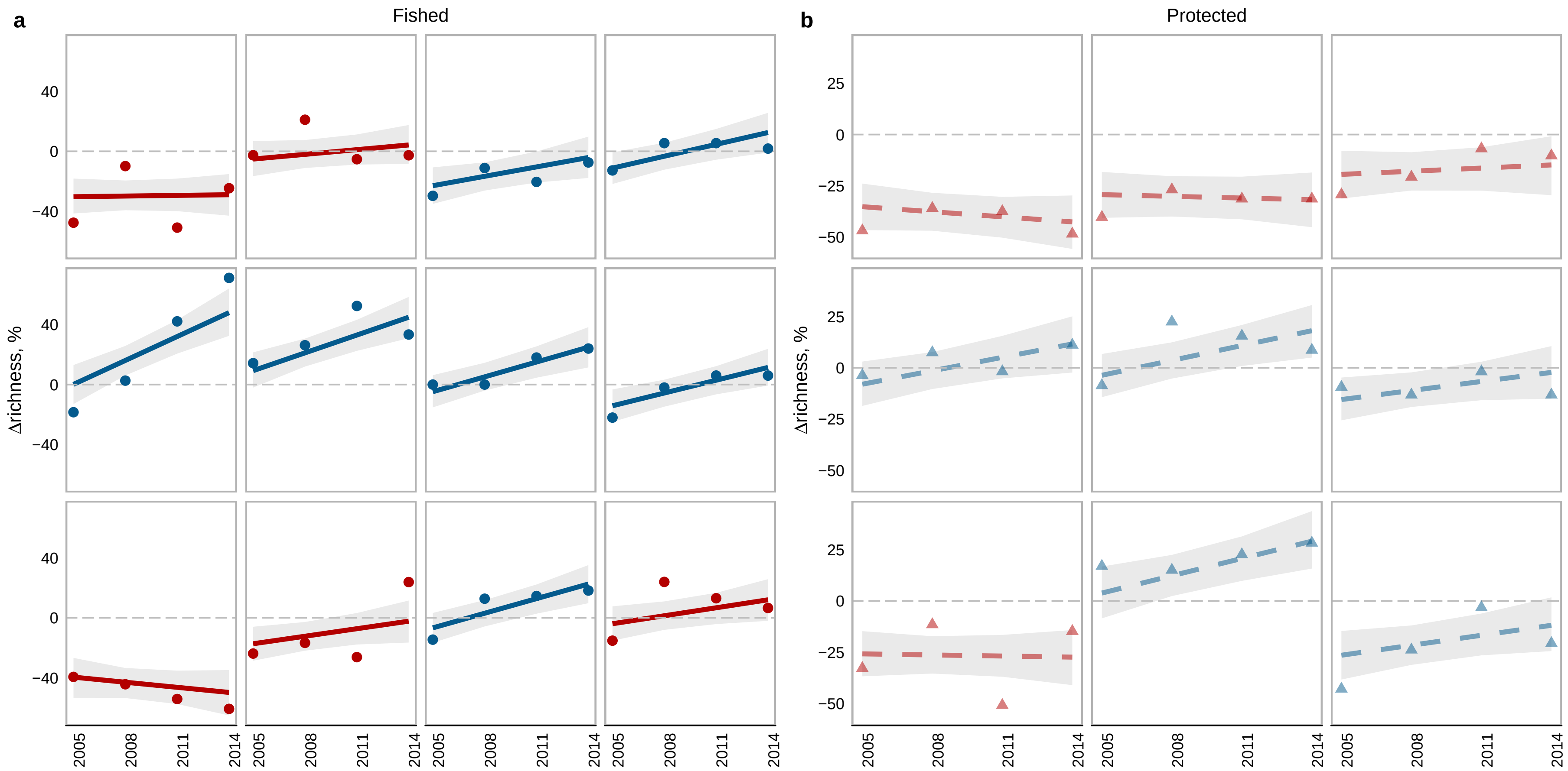

Supplementary Figure 6. Post-bleaching marine reserve effects on species richness of fish assemblages from 2005-2014 for individual reef sites. Panels show the recovery of species richness relative to pre-bleaching levels ($y = 0$, dashed grey line), for fished (a) and protected (b) reefs. Lines are posterior median predictions for each reef site ($\pm$ 95% certainty intervals), for recovering (blue) and regime-shifted (red) habitats. Points are observed data (n sites = 7 fished-recovering, 5 protected-recovering, 5 fished-shifted, 4 protected-shifted).

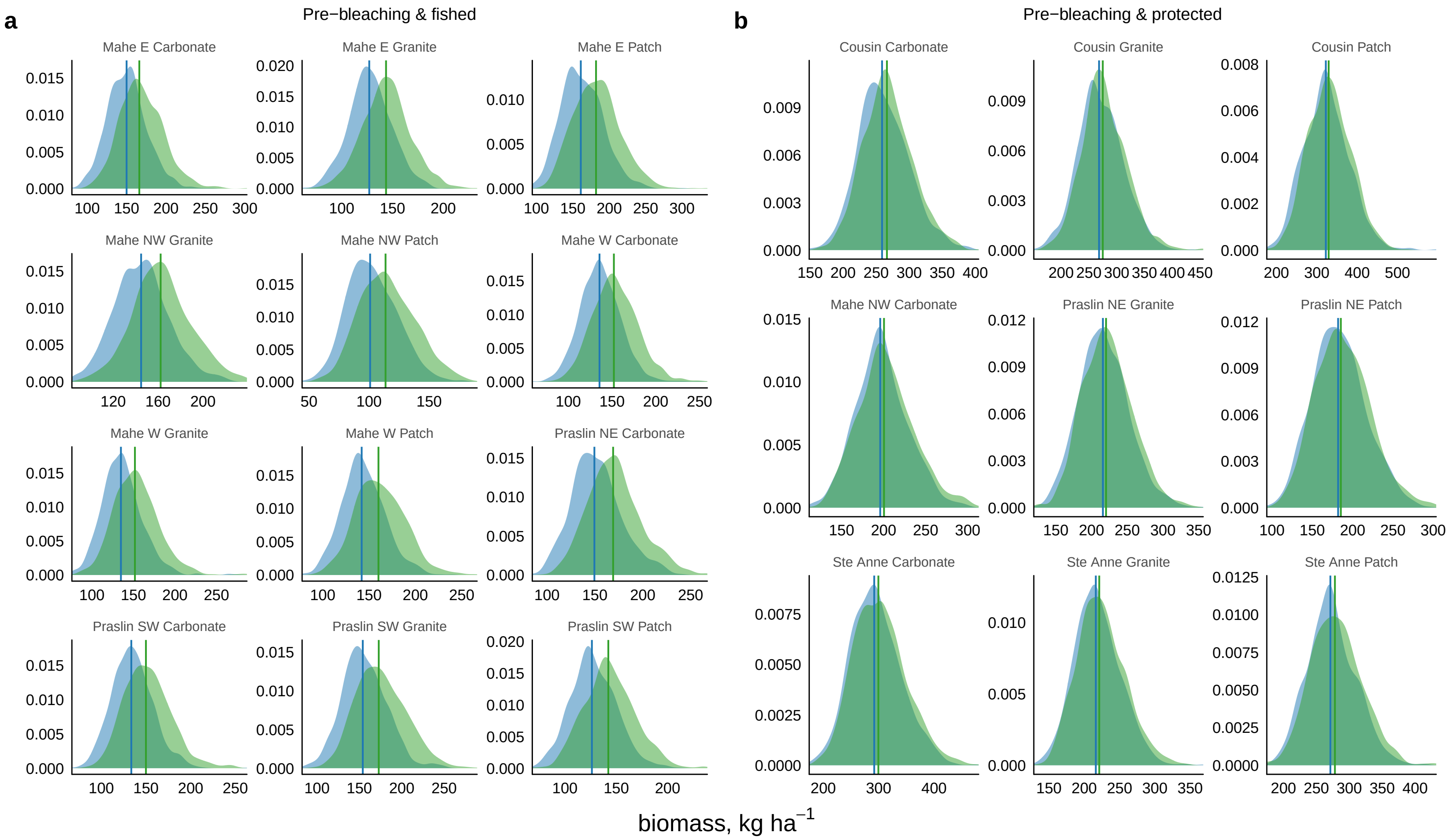

Supplementary Figure 7. Posterior distributions of carnivore and herbivore biomass before coral bleaching at each reef site. For fished (a) and protected (b) reefs, density curves are the posterior distributions of carnivore (blue) and herbivore (green) biomass before bleaching (1994 surveys), with vertical lines indicate median biomass and labelled with site names (y-axis = density).

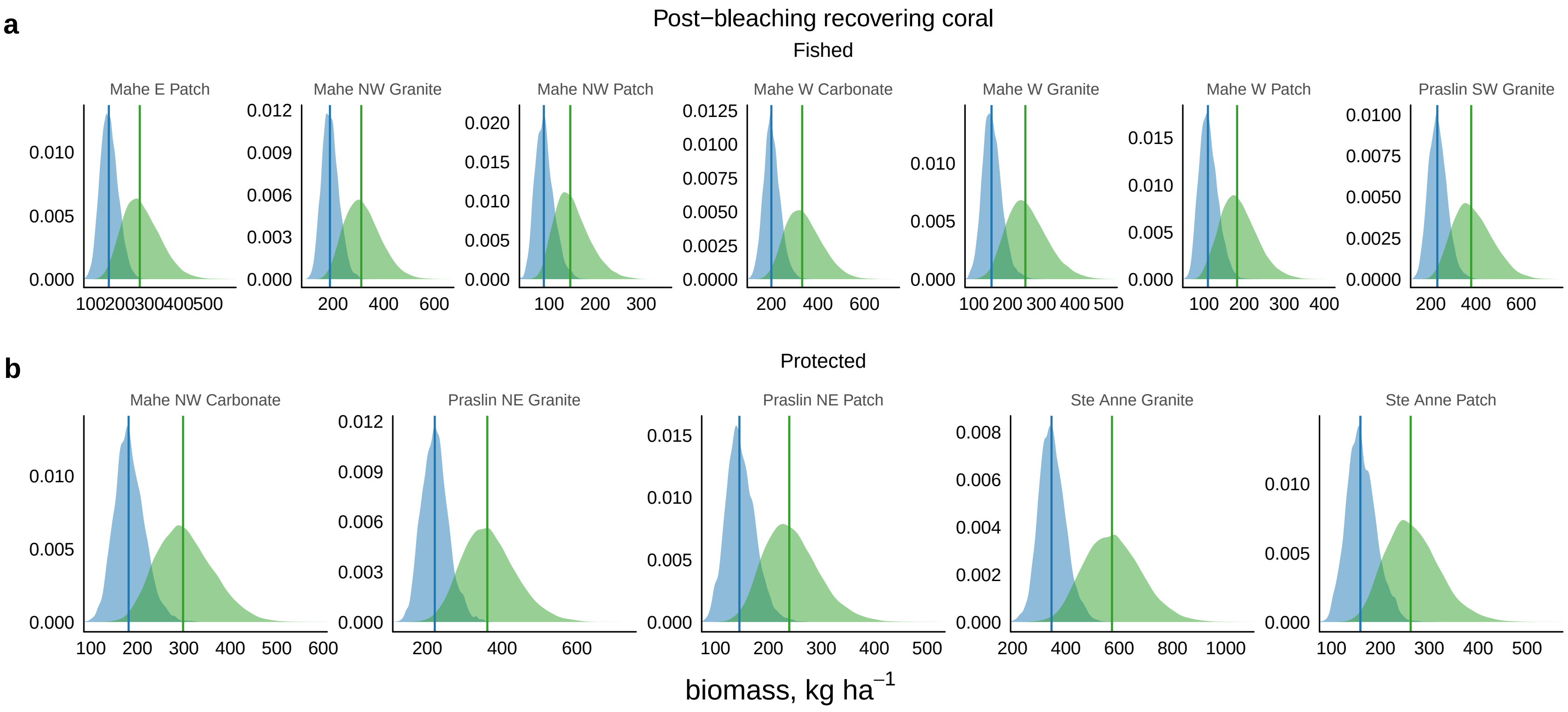

Supplementary Figure 8. Posterior distributions of carnivore and herbivore biomass after coral bleaching on recovering reefs. For fished (a) and protected (b) reefs, density curves are the posterior distributions of carnivore (blue) and herbivore (green) biomass after bleaching (2005-2014 surveys), with vertical lines indicate median biomass and labelled with site names (y-axis = density).

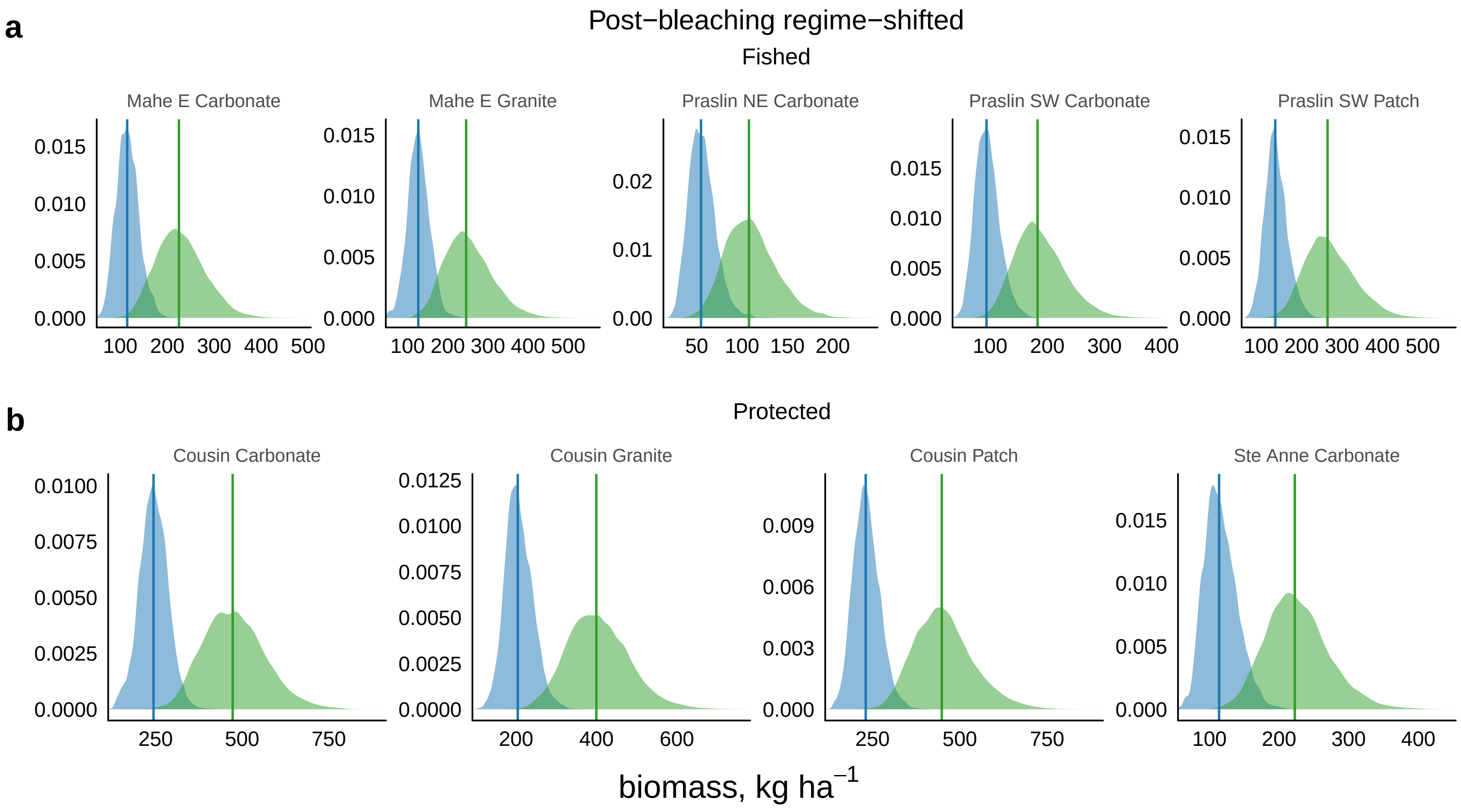

Supplementary Figure 9. Posterior distributions of carnivore and herbivore biomass after coral bleaching on regime-shifted reefs. For fished (a) and protected (b) reefs, density curves are the posterior distributions of carnivore (blue) and herbivore (green) biomass after bleaching (2005-2014 surveys), with vertical lines indicate median biomass and labelled with site names (y-axis = density).

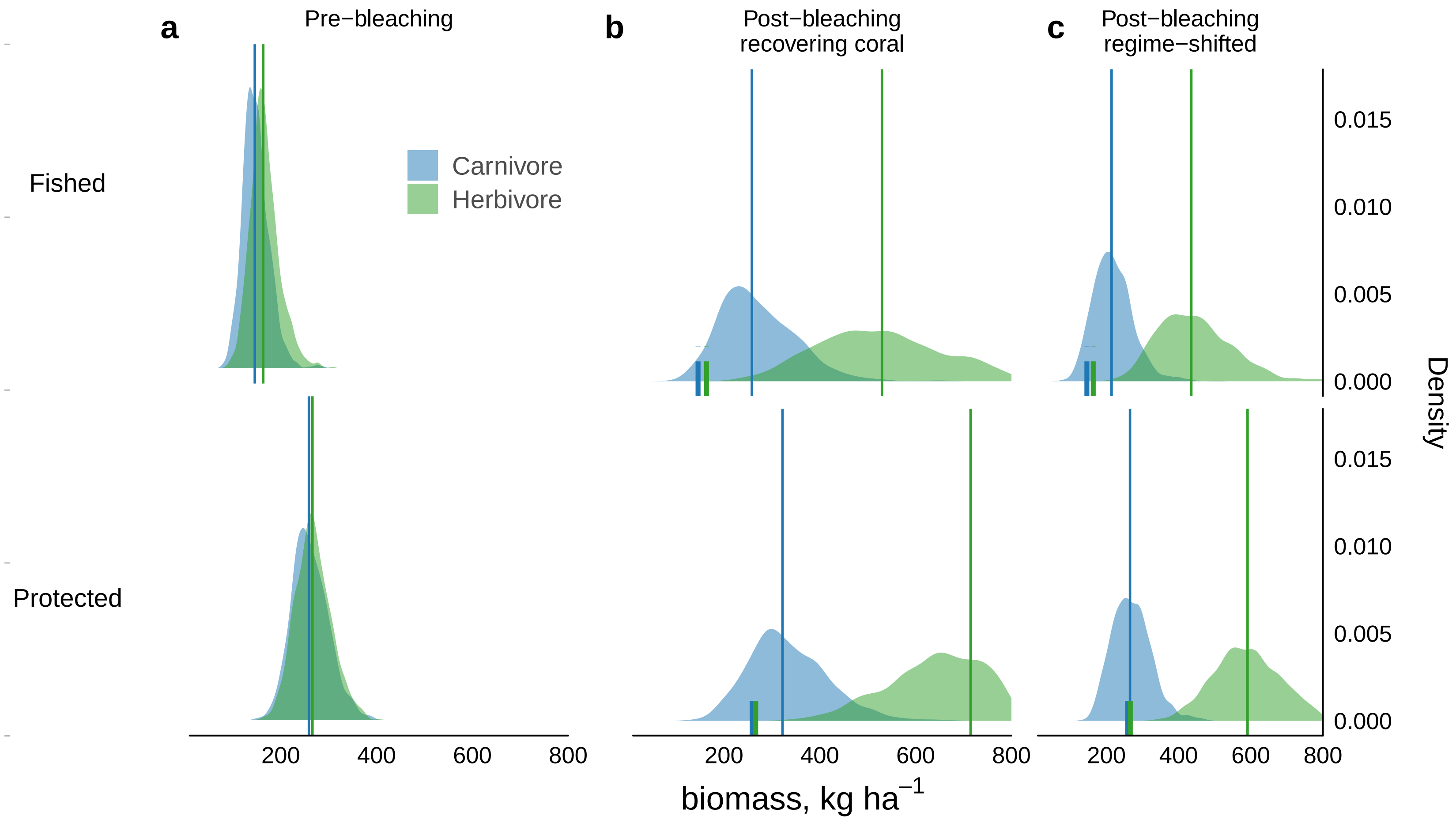

Supplementary Figure 10. Posterior distributions of carnivore and herbivore biomass after coral bleaching on regime-shifted reefs, including 2017 survey data. Marine reserve effects on carnivore and herbivore biomass before and after coral bleaching. For fished (top) and protected (bottom) reefs, density curves are the posterior distributions of carnivore (blue) and herbivore (green) biomass before (a) and after bleaching for recovering coral (b) and regime-shifted (c) reefs. Vertical lines indicate median biomass, with short dashed lines indicating median 1994 biomass on post-bleaching panels (b, c).

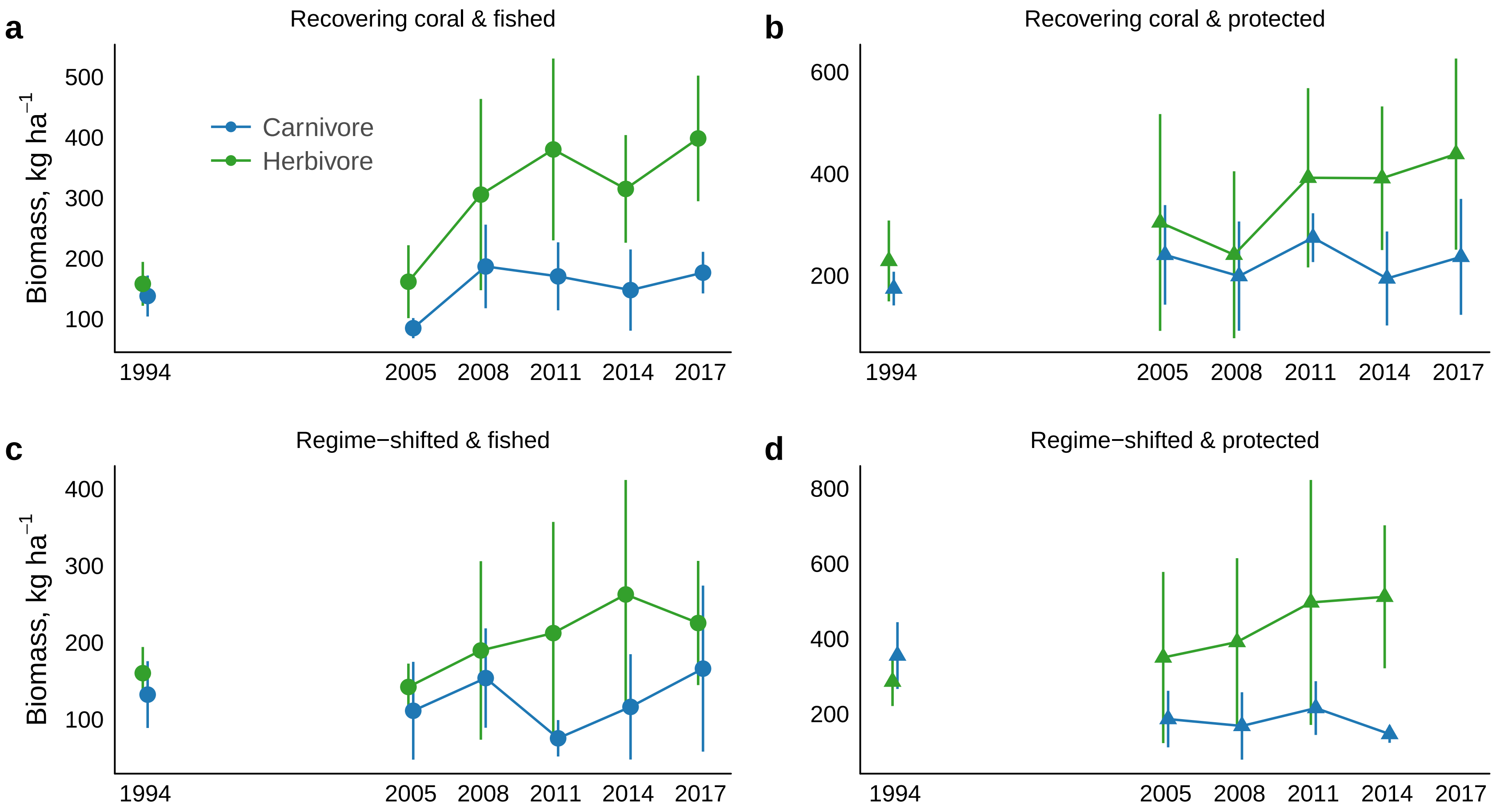

Supplementary Figure 11. Carnivore and herbivore biomass over 1994-2017 on recovering (a, b; top) and regime-shifted (c, d; bottom) reefs, for fished (left, circles) and protected areas (right, triangles). Points are mean biomass (± 2 SE; n sites = 7 fished-recovering, 5 protected-recovering, 5 fished-shifted, 4 protected-shifted). Note 3 regime-shifted & protected reefs were not sampled in 2017 (d), and so were excluded from our main analyses.

Supplementary Table 1. Posterior means and model diagnostics for biomass and richness models (Methods, Eqs. 1,2). Mean, standard deviation (StdDev), 95% certainty intervals for fixed parameters, with effective samples (n_eff) and Gelman-Rubin convergence diagnostic (Rhat).

Model	Covariate	Parameter	Mean	StdDev	lower 0.95	upper 0.95	n_eff	Rhat
Total biomass	Intercept	B_0	8.937	7.658	-6.271	23.837	12782	1.000
	Shifted regime	B_1	0.613	4.823	-8.376	10.566	19617	1.000
	Year	B_2	2.550	2.095	-1.562	6.641	4865	1.000
	Shifted regime * Year	B_3	4.178	2.246	-0.300	8.404	7141	1.000
	Protection	B_4	-0.305	4.764	-9.471	9.111	18499	1.000
	Protection * Year	B_5	-2.618	2.244	-6.898	1.929	8219	1.000
	σ	σ	46.248	4.753	37.293	55.698	1690	1.001
Species richness	Intercept	B_0	-16.644	5.416	-27.215	-6.069	7580	1.001
	Shifted regime	B_1	6.477	6.080	-5.820	17.918	8173	1.001
	Year	B_2	0.870	0.904	-0.878	2.672	9395	1.000
	Shifted regime * Year	B_3	2.420	1.044	0.342	4.434	9366	1.000
	Protection	B_4	-4.624	6.071	-16.423	7.217	11083	1.000
	Protection * Year	B_5	-1.148	1.006	-3.179	0.812	10671	1.000
	σ	σ	12.744	1.368	10.085	15.400	3695	1.001

Supplementary Table 2. Posterior means and model diagnostics for carnivore/herbivore biomass models (Methods, Eqs. 3-5). Mean, standard deviation (StdDev), 95% certainty intervals, effective samples (n_eff) and Gelman-Rubin convergence diagnostic (Rhat).

Model	Covariate	Parameter	Mean	StdDev	lower 0.95	upper 0.95	n_eff	Rhat
1994 dataset (Eq. 4)	Protection [carnivore]	B_1_fg[1]	0.076	0.625	-1.296	1.493	1890	1.001
	Protection [herbivore]	B_1_fg[2]	-0.014	0.625	-1.476	1.334	1905	1.001
	Intercept [carnivore]	B_0_fg[1]	-0.049	0.913	-2.028	1.689	976	1.005
	Intercept [herbivore]	B_0_fg[2]	0.065	0.912	-1.810	1.915	984	1.005
	Intercept	B_0	4.968	0.916	3.081	6.813	1000	1.004
	Protection	B_1	0.495	0.634	-0.839	1.945	1927	1.001
	Gamma scale parameter	k	14.929	4.801	7.137	24.533	2422	1.001
2005-14 dataset (Eq. 5)	Protection * Year [carnivore]	B_5_fg[1]	-0.039	0.616	-1.328	1.356	1487	1.001
	Protection * Year [herbivore]	B_5_fg[2]	0.022	0.616	-1.198	1.489	1510	1.001
	Protection [carnivore]	B_4_fg[1]	0.024	0.673	-1.522	1.368	1314	1.002
	Protection [herbivore]	B_4_fg[2]	0.015	0.673	-1.497	1.380	1299	1.002
	Shifted regime * Year [carnivore]	B_3_fg[1]	0.026	0.685	-1.327	1.545	589	1.002
	Shifted regime * Year [herbivore]	B_3_fg[2]	0.025	0.690	-1.320	1.541	575	1.003
	Year [carnivore]	B_2_fg[1]	-0.074	0.671	-1.465	1.480	2574	1.001
	Year [herbivore	B_2_fg[2]	0.034	0.669	-1.275	1.681	2550	1.001
	Shifted regime [carnivore]	B_1_fg[1]	0.115	0.756	-1.498	1.824	1916	1.001
	Shifted regime [herbivore]	B_1_fg[2]	-0.050	0.756	-1.600	1.708	1917	1.000
	Intercept [carnivore]	B_0_fg[1]	-0.293	1.621	-3.469	2.566	1204	1.002
	Intercept [herbivore]	B_0_fg[2]	0.377	1.623	-2.760	3.280	1199	1.002
	Intercept	B_0	4.992	1.627	2.129	8.208	1213	1.003
	Shifted regime	B_1	0.148	0.778	-1.563	1.796	1914	1.000
	Year	B_2	0.145	0.670	-1.430	1.518	2549	1.001
	Shifted regime * Year	B_3	0.001	0.689	-1.572	1.297	579	1.003
	Protection	B_4	0.405	0.694	-0.965	1.954	1352	1.002
	Protection * Year	B_5	-0.064	0.616	-1.459	1.234	1496	1.001
	Gamma scale parameter	k	46.763	5.782	36.150	58.324	10836	1.000
