# Supplementary material for: Changing role of coral reef marine reserves in a warming climate
Source: Nat Commun. 2020 Apr 24;11:2000. doi: 10.1038/s41467-020-15863-z (PMC7181733; doi:10.1038/s41467-020-15863-z)
Supplement: Supplementary file 3 — Reporting Summary [file 41467_2020_15863_MOESM3_ESM.pdf]

nature research

Corresponding author(s): Nicholas Graham

Last updated by author(s): Mar 13, 2020

Reporting Summary

Nature Research wishes to improve the reproducibility of the work that we publish. This form provides structure for consistency and transparency in reporting. For further information on Nature Research policies, see Authors & Referees and the Editorial Policy Checklist .

Statistics

For all statistical analyses, confirm that the following items are present in the figure legend, table legend, main text, or Methods section.

n/a	Confirmed	
☐	☒	The exact sample size (n) for each experimental group/condition, given as a discrete number and unit of measurement
☐	☒	A statement on whether measurements were taken from distinct samples or whether the same sample was measured repeatedly
☐	☒	The statistical test(s) used AND whether they are one- or two-sided<br>*Only common tests should be described solely by name; describe more complex techniques in the Methods section.*
☐	☒	A description of all covariates tested
☐	☒	A description of any assumptions or corrections, such as tests of normality and adjustment for multiple comparisons
☐	☒	A full description of the statistical parameters including central tendency (e.g. means) or other basic estimates (e.g. regression coefficient) AND variation (e.g. standard deviation) or associated estimates of uncertainty (e.g. confidence intervals)
☒	☐	For null hypothesis testing, the test statistic (e.g. F, t, r) with confidence intervals, effect sizes, degrees of freedom and P value noted<br>*Give P values as exact values whenever suitable.*
☐	☒	For Bayesian analysis, information on the choice of priors and Markov chain Monte Carlo settings
☐	☒	For hierarchical and complex designs, identification of the appropriate level for tests and full reporting of outcomes
☐	☒	Estimates of effect sizes (e.g. Cohen's d, Pearson's r), indicating how they were calculated

Our web collection on statistics for biologists contains articles on many of the points above.

Software and code

Policy information about availability of computer code

Data collection	No software was used for data collection
Data analysis	Analyses were conducted in R 3.6.0, and Bayesian hierarchical models were implemented in Stan 2.18.1 using rethinking and RStan

For manuscripts utilizing custom algorithms or software that are central to the research but not yet described in published literature, software must be made available to editors/reviewers. We strongly encourage code deposition in a community repository (e.g. GitHub). See the Nature Research guidelines for submitting code & software for further information.

Data

Policy information about availability of data

All manuscripts must include a data availability statement. This statement should provide the following information, where applicable:

- Accession codes, unique identifiers, or web links for publicly available datasets
- A list of figures that have associated raw data
- A description of any restrictions on data availability

We provide data and R code associated with this study at an open source repository (github.com/jpwrobinson/changing-mpas)

Field-specific reporting

Please select the one below that is the best fit for your research. If you are not sure, read the appropriate sections before making your selection.

☐ Life sciences ☐ Behavioural & social sciences ☒ Ecological, evolutionary & environmental sciences

For a reference copy of the document with all sections, see nature.com/documents/nr-reporting-summary-flat.pdf

Ecological, evolutionary & environmental sciences study design

All studies must disclose on these points even when the disclosure is negative.

Study description	The study aimed to assess how the ecological responses to marine protected areas on coral reefs changed following major climate-disturbance. This was achieved through analyses of a long-term ecological dataset of 21 coral reefs in the inner Seychelles, 9 of which are in marine protected areas, and 12 are in nearby openly fished areas.
Research sample	The reef benthos, and associated fish communities, were surveyed using underwater visual census techniques. At each of the 21 sampled reefs, 8 to 16 stationary point counts were conducted. Point counts were 7m radius (154m2 in area), and separated by at least 15 m. This sampling method was established at the start of the time series (1994) and is widely used in coral reef surveys.
Sampling strategy	The number of reefs surveyed was chosen at the start of the dataset (1994) to provide good coverage of the entire range of the inner islands of the Seychelles, and to include multiple marine protected areas and fished areas. Underwater visual surveys (n=8-16 per reef) followed standard replication numbers for these methods from other studies, and power analyses have indicated that this level of replication is sufficient to detect changes in these data.
Data collection	Fish abundance and individual body length (to the nearest cm) of diurnally active non-cryptic species (134 species from 16 families) were estimated in each point count area. The percent cover of live hard coral, soft coral, macroalgae, sand, rubble, and rock was quantified visually and using 10m line intercept transects in each point count area. The structural complexity of the reef was visually estimated on a 6 point scale. Simon Jennings collected 1994 data. Nicholas Graham collected all fish survey data 2005-2017. Shaun Wilson collected all benthic survey data 2005-2017.
Timing and spatial scale	The sampled reefs span the entire range of the inner islands of the Seychelles, that cover an area of approximately 2500 km2. Surveys were first conducted in 1994. Following the major coral bleaching event of 1998, the reefs were re-surveyed in 2005. The reefs have been re-surveyed once every 3 years since 2005, to capture change through time, but allow enough time between sample collection periods for changes to be detectable.
Data exclusions	No data was excluded from the analyses
Reproducibility	A full description of the methodologies used is provided in the Methods, and the data and full code necessary to reproduce the findings are provided through the GitHub link in the Methods. All attempts at replication were successful.
Randomization	Underwater visual survey counts were started at a random point close to the GPS marker for the site, and at the depth contour surveyed for that site. Each count replicate was separated by a minimum of 15m. There was no randomization or organisms into treatment groups as these are observational field data.
Blinding	Blinding was not necessary as these were surveys of wild communities of benthic organisms and fish.

Did the study involve field work? ☒ Yes ☐ No

Field work, collection and transport

Field conditions	Field (sea) conditions were calm.
Location	The study was conducted on the shallow (3-9m depth) fringing reefs of the inner Seychelles Islands (-4.711 S, 55.573 E)
Access and import/export	All surveys were conducted with research permit clearance from the Seychelles Bureau of Standards.
Disturbance	All surveys were observational only, and no disturbance was caused.

Reporting for specific materials, systems and methods

We require information from authors about some types of materials, experimental systems and methods used in many studies. Here, indicate whether each material, system or method listed is relevant to your study. If you are not sure if a list item applies to your research, read the appropriate section before selecting a response.

Materials & experimental systems

n/a	Involved in the study	
☒	☐	Antibodies
☒	☐	Eukaryotic cell lines
☒	☐	Palaeontology
☐	☒	Animals and other organisms
☒	☐	Human research participants
☒	☐	Clinical data

Methods

n/a	Involved in the study	
☒	☐	ChIP-seq
☒	☐	Flow cytometry
☒	☐	MRI-based neuroimaging

Animals and other organisms

Policy information about studies involving animals; ARRIVE guidelines recommended for reporting animal research

Laboratory animals	The study did not involve laboratory animals.
Wild animals	All wild animal work was observational (visual surveys).
Field-collected samples	The study did not involve samples collected from the field.
Ethics oversight	No ethical clearance was necessary as the study was observational.

Note that full information on the approval of the study protocol must also be provided in the manuscript.
